# Farther Remarks on the Method of Treating the Remittent Fevers of the West Indies; with Observations on the Best Means of Preserving Health in Jamaica

**Published:** 1782

**Authors:** 


### SECTION II.

### ESSAYS AND OBSERVATIONS.

I. *Farther Remarks on the Method of treating the remittent Fevers of the Weſt Indies; with Obſervations on the beſt Means of preſerving Health in Jamaica. By* Dennis Ryan, M. D. *In a Letter to* Robert Adair, *Eſq; Inſpector-General of Hoſpitals. Communicated by* Dr. Simmons, F.R.S. *Read* Jan. 7, 1782.

I Have formerly had occaſion, in a letter to my worthy friend Dr. Simmons, to mention ſome of the principal circumſtances that diſtinguiſh the fevers of this country from thoſe which prevail in Europe, and have taken notice of the particular treatment which ſuch peculiarities ſeemed to require.* I have in general obſerved, even in the moſt violent inſtances, not excepting thoſe which are attended with a yellowneſs of the ſurface of the body, that the fe-

* See our Journal for October 1781.

vers vers here feldom affume the proper continued type; and that, unlefs the patient is carried off by the firft attack, an evident remiffion commonly takes place; fo that in nine cafes out of ten they may be faid to come under the denomination of remittents. In the beginning they are for the moft part attended with fome inflammatory fymptoms, but thefe are feldom fo fevere as to make bleeding neceffary, except in perfons of full and robuft habits. A vomit given in the very commencement of the difeafe may be of great fervice in difcharging the foul and bilious contents of the ftomach, but at a more advanced period a conftant retching renders its ufe often precarious, and fometimes inadmiffible. It is however, always proper to obtain a fpeedy evacuation by the inteftines. The patients fhould get oranges in abundance. In general they wifh for them, and befides their refrigerant and laxative effects, where the vomiting is troublefome, they often put a ftop to it, and fupercede the employment of opiates for that purpofe. For common drink, a beverage made with lime-juice, water, and a little fugar, feems to be the beft and moft grateful; nor have I feen any difadvantage arife from indulging the patient accooccaſionally with draughts of cold water. Antimonials, when the ſtomach is not too irritable, and warm fomentations to the abdomen and lower extremities, are other means proper to be employed in this ſtage of the diſorder. But where there occurs a violent delirium, great benefit may be obtained from the uſe of a bliſter, or by drawing ſome blood from the temples by means of cupping-glaſſes, or from the arm, if the patient's ſtrength will allow it.

About the third day, and ſometimes earlier, a remiſſion occurs, and, ſoon after, ſymptoms of debility manifeſt themſelves; or as ſome writers expreſs it, too weak a re-action takes place. Nature becomes too feeble to combat the diſeaſe, and calls for ſupport and aſſiſtance. To anſwer this end, in ſome countries much confidence ſeems to be put in the uſe of contrayerva, ſerpentaria, and other cordial medicines. Thus at *La Charité*, a celebrated hoſpital at Paris, I have frequently ſeen inſtances of low nervous fevers, in which the phyſicians contented themſelves with preſcribing the *potio cardiaca minor*; and when great loſs of ſtrength, and perhaps involuntary diſcharges threatened danger, the *potio cardiaca major* was ſubſtituted for it. But in this climate, theſe remedies ſhould never be employed except it be to aſſiſt the operation, or ſupply the want, of others which are more powerful and certain in their effects. As ſoon then as the remiſſion takes place, we muſt have immediate recourſe to the bark, in ſubſtance. And, as I have obſerved elſewhere, ſome caſes will require it in decoction even before the fever has abated, whilſt in others the ſymptoms are ſo urgent, that at firſt ſight you muſt give it in ſubſtance, as the delay of giving a vomit or a purge may prove fatal to the patient. The rule is, to give the bark in as great a quantity as the ſtomach can bear. The uſe of oranges ſhould be ſtill continued, for they tend to make the bark ſit eaſy on the ſtomach, and keep the body open. Where they have not this effect, ſmall doſes of ſal polychreſt will be occaſionally neceſſary. If on the contrary the bark ſhould occaſion a purging, it muſt be combined with opiates. If the ſtomach will not retain it, it muſt be employed in glyſters and fomentations.

I believe it may be laid down as a rule, that in moſt caſes of fever, where the bark is neceſſary, wine is likewiſe proper. But though the practitioners in this country are in general unaniunanimous as to the importance of the bark, yet that does not ſeem to be the caſe with reſpect to the uſe of wine. I happened once to be preſent when two or three of the moſt reſpectable phyſicians in the iſland held a conſultation on the caſe of a patient, who, beſides other dangerous ſymptoms, was affected with a coldneſs of the upper and lower extremities. They ordered him the bark in great quantity, but ſeemed to lay no ſtreſs on the uſe of wine. However, I cannot help giving it as my opinion, that in ſuch caſes, where the powers of life are evidently ſunk and oppreſſed, there is reaſon to apprehend that the bark will remain an inactive load on the ſtomach, and that the employment of good wine is the moſt expeditious and effectual means of giving a ſtimulus to the ſyſtem, and recovering the patient from the danger with which he is threatened. Some of the patients whom I had under my care recovered after the occurrence of the ſymptoms above mentioned, which I am inclined to attribute to the liberal uſe of wine, as much, if not more than to the effects of the bark. And I muſt own that I have found an obſervation, which I heard from my learned maſter, Dr.

Cullen, always true and well founded, viz. that wine is of little ſervice unleſs it be given in large quantity. I know ſome medical gentlemen, who ſay, that about half a pint of wine a day is ſufficient for any ſoldier in an hoſpital: but I think I have ſaved the lives of ſeveral ſoldiers, by allowing them eight times that quantity. Dr. Cullen mentions a caſe of Typhus where, I think, the patient was ſaid to have taken ſeven bottles of claret in the ſpace of twenty-four hours; but the doctor doubts whether the nurſe may not have aſſiſted him. Whatever may be the caſe in private practice, I believe, that in public hoſpitals of every kind, the orderly-men and nurſes are too apt to think that the patients may diſpenſe with leſs wine than is ordered for them; and hence they are tempted to make free with their allowance. This I have known to be the caſe; and as it cannot eaſily be prevented, it is, I think, a circumſtance that ſhould be had in view, when wine is preſcribed. It is better to make ſome allowance for the nurſe or orderly-man, who if they do their duty, have no ſmall hardſhip to go through, than that the life of the patient

ſhould be loſt, from his being ſtinted in what is judged neceſſary for him.

Notwithſtanding what I have ſaid in favour of wine, I muſt own that I have met with ſome few caſes, wherein, though a ſlight remiſſion afforded an opportunity of giving the bark, ſtill ſymptoms of irritation and phlogiſtic diatheſis rendered it adviſeable to be ſparing and cautious in the uſe of wine. But a man may always proceed with ſafety in ſuch caſes, by taking care to judge of the propriety of the meaſures, which he has adopted, only by the effects which they produce. Thus if I find that wine has made the pulſe full and ſteady, from being feeble and quick; that it has diminiſhed the febrile heat and anxiety, and removed, inſtead of increaſing, any delirium that might have been preſent, ſo as to bring on a certain ſerenity of mind; if it occaſions a gentle moiſture on the ſkin, if it removes thirſt, and laſtly, if the patient has a craving deſire for it, I have no doubt but I ſhould perſiſt in its uſe. But if the effects which it produces be oppoſite to thoſe juſt now mentioned, it is evidently contraindicated. It may be aſked whether it be neceſſary to give the bark in every caſe of proper fever in this country? In anfwer to this queftion, I muft own that in the military hofpitals I have met with a few patients, whofe fevers began in the ordinary manner, but during the remiffions, either through their diflike of the bark, or the neglect of the orderly-men, this remedy was not employed; notwithftanding which they recovered. Befides, in fome particular habits the bark, although given only in the time of remiffion, feems to caufe great irritation, anxiety, and quicknefs of pulfe. I remember to have feen an inftance of this kind. The firft paffages were evacuated, and fome blood taken away, and as there were appearances of danger, it was thought proper as foon as a remiffion took place, to try the bark in a large quantity, but the patient had fo great an averfion to it, that he could not be prevailed on to take it, and the anxiety which followed his fwallowing a dofe of it, together with the efforts he made to throw it up again, may well have arifen from this difguft. Notwithftanding this, after a very fevere difeafe, he recovered, and thanked his ftars that he efcaped by following his own inclinations in fpite of the doctors.

Some time laft December, I was myfelf affected with the ufual figns of the beginning of a fever, attended with a fenfation of prickling cold all over my body. I was fhortly after feized with a head-ach, confiderable heat, and quicknefs of pulfe. Thefe fymptoms continued to grow worfe as the day declined. At night I took an emetic which difcharged a quantity of bile; but the febrile fymptoms continued as before, and were accompanied with great reftleffnefs and anxiety. The following day a mixture was made up for me, compofed of three pints of lemonade, in which an ounce of fal polychreft, and five grains of tartar emetic, had been diffolved. Of this I took about a tea cup full every two hours, and in the intervals made ufe of fage and balm tea. In eight or ten hours the mixture operated by the inteftines, and brought on a gentle diaphorefis; fo that on the beginning of the third day I had a diftinct remiffion from the fever. Dr. Ledwich, a fkilful phyfician in Kingfton, who attended me, immediately propofed the bark. But however liberal I was of it to others, I had fuch difguft to it myfelf that I intreated, and perfuaded him to defer employing it, till after another exacerbation; but the fever did not return, though

 the the effects of the firft attack were fo fevere that I was confined to my room for feveral days.

It appears then that there are fome cafes of fever in which the bark is not neceffary. And I have been informed by Dr. Prendergaft, an eminent phyfician, who has practifed with great reputation for fome years in Kingfton, that inftances of this kind moftly occur among the natives, and fuch as have refided for fome time in the country, both of whom, he fays, are fometimes of fuch inflammatory habits, as to bear repeated bleedings. But as cafes of this kind are rather rare, and as the fymptoms by which they may be known in the beginning from others of a more dangerous tendency, are not yet fully afcertained, it feems proper and neceffary to try the bark in every cafe, as foon as a remiffion occurs. To act otherwife is imprudent, as it is beyond doubt, that in all hot climates, thoufands die for want of this valuable remedy; and as it is equally certain, that a patient feldom or never dies in confequence of having taken it under proper management.

Several rules have been laid down, for preferving the health of ftrangers on their arrival here; but fome of them, I think, are abufed, and the reſt are but little attended to. When people are expoſed from their ſituation to malignant putrid diſorders, and a foul infectious air, the peruvian bark has been juſtly recommended as a prophylactic, and I make no doubt but the uſe of it in this country, during the ſickly months, may be attended with great advantage in ſome conſtitutions, but it ſhould be employed with caution and moderation. I know people here who, on the appearance of any indiſpoſition, immediately ſwallow half an ounce, or even a whole ounce of bark in ſubſtance, and repeat it as they think occaſion requires. I cannot, however, help thinking that there are few who would derive any benefit from this practice, I believe I may even venture to ſay, that there are but few to whom it would not prove prejudicial. Fevers, in this climate, are allowed to begin in general with inflammatory ſymptoms; now as the bark is poſſeſſed in an eminent degree of the powers of increaſing the tone and vigour of the ſanguiferous ſyſtem, it is evident, that when taken in this manner, it will have a tendency to make the firſt attack of the fever more dangerous and violent than it otherwiſe might be. Beſides this, it may have another another bad effect in binding the body, and caufing an accumulation of bile, which never fails to aggravate the fymptoms of every difeafe in warm climates: and when a man accuftoms himfelf to take fo powerful a remedy, as the bark is, in great quantity, at a time that he does not ftand in real need of it, he has reafon to expect the lefs advantage from it, when his life may be in danger, and its ufe becomes effentially neceffary. To prevent then any evil arifing from what is intended as a remedy, it may not be improper to confine thofe who take bark as a prophylactic, to a glafs or two of an infufion of it, to be taken every morning, and thofe who feem chiefly to require it, are fuch as are of feeble, languid habits, and have been formerly liable to remittent or intermittent fevers.

Temperance, and a ftrict adherence to a regular mode of living, is another of the means recommended for the prefervation of health in this climate. But it muft be owned that the inhabitants of this ifland live as freely as if the advice had been, 'veneri,vino, gulæ indulgeatur.' I have feen no country where people in general are of a more fociable and friendly difpofition, and and however they may differ from each other in political matters, they are firmly united as members of civil ſociety. The pleaſure with which they enjoy the gifts of fortune, and the fruits of their induſtry, is proportioned to the number of thoſe whom they make partakers of them. And that hoſpitality for which Ireland, and ſome parts of Great-Britain were formerly renowned, ſeems now to have fixed its reſidence in this country. But it is to be obſerved, that theſe obſervations apply leſs to Kingſton, the inhabitants of which are almoſt all in a commercial line, than to any other part of the iſland. Where a ſpirit of ſociety and hoſpitality thus prevails, people are ſeldom very abſtemious; for when men come together with an intention to forget their cares and be happy, they naturally employ every means that have the effect of making them believe they are ſo. And as it is well known that good cheer and good liquor anſwer this end, it is an eſtabliſhed cuſtom here to have recourſe to them on all ſuch occaſions. Even the ſpiritual and medical adviſers of temperance and regularity, are themſelves infected with the love of this kind of ſociety and good living; and the old old phrafe,—Do as I fay, but don't do as I do, is no where more neceffary.

It is however furprifing to fee what good health people enjoy who live almoft continually in this unreftrained manner;—and what is more ftrange, I have feen feveral who, on their arrival from Europe, have purfued the fame meafures with impunity. One could not from reafon and fpeculation promife them fuch length of days as they frequently enjoy. There has been lately inftituted at Kingfton a club, or fociety, called the European Club, of which no man can be a member that has not been thirty years refident in the ifland. The great number who within this month have attended this meeting, and the ftill greater number, who though qualified could not be prefent, on account of the diftance, leave us no room to doubt that many Europeans live to a good old age in Jamaica; and the very copious libations of claret with which they celebrate this feftival, plainly tell us that they are no water-drinkers, and that it is not by abftemioufnefs they have got the better of this climate.

I am aware that fome people may be apt to conclude from this account, that on their arrival here they may fet out without reftraint, and live live as they see others do; but such a conclusion would be altogether unjust, and might prove pernicious. For though the inhabitants have by long habit inured themselves to drink and live freely, without any apparent ill consequence, it would be exceedingly dangerous for strangers at once to adopt their example. I have, indeed, just observed, that some strangers have done so with impunity; but they were such as had been hardened in the same habit before they left Europe. Others must proceed by slow and cautious steps, and run many risques before they can arrive at the security I speak of. And I am confident that many from too great impatience to accomplish their wishes, lose their lives in the practice of those very measures, which are necessary to attain it. I cannot then hesitate still to recommend temperance as the surest and safest means of preserving the health, particularly of strangers, in this, or any other part of the West-Indies. It is more especially necessary for such as happen to be any way sickly or infirm: though as most people come here more with a view to mend their fortunes than their constitutions, there are but few in that situation on their arrival: on the contrary, they are in general general ſuch as would be able to bear a great deal, if they could be perſuaded to live within due bounds of moderation, and reſiſt the wrong and imprudent counſels of thoſe who themſelves tranſgreſs them. I know ſeveral gentlemen in this iſland, who live happy, though in a very moderate and abſtemious manner. And the ladies who commonly drink nothing but water, enjoy the beſt health.

Beſides avoiding exceſs in drinking, and free living in general, ſtrangers ſhould accuſtom themſelves to early riſing, and ſea-bathing, where it is convenient. They ſhould not expoſe themſelves too much to the ſun, or to the night-winds, when they are over heated. They ought to take frequent but moderate exerciſe in the cool of the day, and refrain from meat ſuppers, and what are called here ſecond breakfaſts. They ſhould be particularly careful to keep their bodies open by the uſe of fruit and vegetables, or other means, if neceſſary; as nothing is more unfavourable to health in this climate than a coſtive habit. I muſt alſo caution them againſt a kind of medley, which is drank here by way of cordial, between breakfaſt and dinner: it is compoſed of rum or brandy, mixed with milk, water, nutmeg, and ſugar, and from ſome ſalutary effects it is ſuppoſed to produce, it has got the name of *doctor*,—but it ſhould rather be ranked among the *quacks*, and ſo doomed to fall into the diſuſe and contempt it deſerves.

In other reſpects, thoſe who enjoy good health, ſhould not be over anxious about themſelves, or think it neceſſary to dabble with medicines. But even though ſurrounded on every ſide by death and ſickneſs, they ſhould preſerve a conſtant gaiety and chearfulneſs of mind; and not allow the apprehenſions of future danger to embitter the enjoyment of preſent happineſs.—They ſhould follow that excellent advice of Celſus: "Sanus homo qui et bene valet, et ſuæ ſpontis eſt, nullis obligare ſe legibus debet; ac neque medico neque iatroalipta egere. Hunc oportet varium habere vitæ genus: modo ruri eſſe, modo in urbe, ſæpiuſque in agro: navigare - - - - quieſcere interdum, ſed frequenter ſe exercere."*

I ſend you theſe obſervations, ſuch as they are, from a deſire I have that the medical hiſtory of this iſland may be better known than it is at

* Lib. i. cap. 1.

present present, and I hope that your indulgence and approbation of my feeble essays, may encourage others, who have more leisure and abilities, to pay attention to the same subject, and be more full and satisfactory in their accounts.

*Jamaica,*
*April* 24, 1781.

